# Development of a predictive nomogram for postembolization syndrome after transcatheter arterial chemoembolization of hepatocellular carcinoma

**DOI:** 10.1038/s41598-024-53711-y

**Published:** 2024-02-08

**Authors:** Jinfeng Bai, Ming Huang, Jinmei Zhou, Bohan Song, Jianjie Hua, Rong Ding

**Affiliations:** Minimally Invasive Intervention Department, The Third Affiliated Hospital of Kunming Medical University, Kunming, 650118 China

**Keywords:** Hepatocellular carcinoma, Transcatheter arterial chemoembolization, Post embolization syndrome, Gastroenterology, Oncology

## Abstract

Post-embolization syndrome (PES) is a frequent complication after receiving transcatheter arterial chemoembolization (TACE) in patients with hepatocellular carcinoma (HCC), but only a few studies have focused on the factors influencing PES in those patients. In this study, the impact factors of PES were explored and a nomogram was constructed to predict the occurrence of PES in HCC patients with TACE. This was a retrospective cohort study of HCC patients who underwent TACE obtained from the third affiliated Hospital of Kunming Medical University between January 1, 2020, and September 1, 2022. T‑test and Chi‑square test were used to search for factors influencing PES occurrence, and then the nomogram was further established based on multivariable logistic regression analysis. Validation of the predictive nomogram was also evaluated by calibration curve, concordance index (C-index), and receiver operating characteristic (ROC) curves. The enrolled patients (n = 258) were randomly assigned to the primary cohort (n = 180) and validation cohort (n = 78) in a 7:3 ratio. Among 180 patients in the primary cohort, 106 (58.89%) experienced PES. TACE types (*P* = 0.015), embolization degree (*P* = 0.008), and tumor number (*P* = 0.026) were identified as predictors by the logistic regression analysis and were used to develop the predictive nomogram. The internally validated and externally validated C-indexes were 0.713 and 0.703, respectively. The calibration curves presented good consistency between actual and predictive survival. Types of embolic agents, embolization degree, and tumor number were found to be the predictors of PES after TACE. The nomogram could reliably predict PES in HCC patients with TACE. This predictive model might be considered for clinical practice.

## Introduction

Hepatocellular carcinoma (HCC) is the most frequent type of primary liver cancer. It is the third most common cause of cancer-related deaths, with an estimated 782,500 new cases each year and increasing mortality^1,2^. The majority of patients are diagnosed with HCC at a moderate-to-advanced stage, and only a small percentage of patients are eligible for radical treatments, including liver transplantation and surgical resection^3,4^. For patients who are unfit for surgery, transcatheter arterial chemoembolization (TACE) is regarded as a primary treatment. A recent report has shown that for patients with advanced HCC, TACE increases median survival from 16 to 20 months^5^; however, many patients still complain of some adverse reactions. The most frequent complications after receiving TACE are post-embolization syndrome (PES), including transient abdominal pain, nausea, vomiting, and fever. The duration of PES caused by TACE is self-limited, but it often results in prolonged hospitalization and postoperative costs. In addition, PES also represents a negative therapeutic experience for HCC patients.

Currently, only a few studies have explored the risk factors affecting the development of PES in patients with HCC after TACE. However, these risk factors include differences in prevalence between studies, which may be caused by different sample sizes or inclusion criteria. Besides, no corresponding clinical prediction model has been developed, making it impossible to achieve accurate prediction of PES. Nomograms have been deemed a reliable and visible prediction tool, which is beneficial for important prognosis studies and risk assessment of cancer patients^6–8^. The traditional regression model can be undoubtedly conveniently and accurately transformed into a visual risk estimation for each patient. In this way, clinicians can vividly show patients their predictions of future events rather than roughly report corresponding risk factors, which plays a positive role in improving patient compliance. In addition, according to the model, clinicians can prospectively identify which patients are at risk for the development of post-TACE PES and which patients could withstand early discharge. Based on this model, an appropriate therapeutic strategy could be selected individually.

In this study, we explored relevant factors that affect PES after TACE and their interrelationship. Moreover, statistical tools were applied to create and validate comprehensive predictive models of PES in patients with HCC treated with TACE. This model is expected to prospectively identify patients who are at high risk for PES.

## Materials and methods

### Data source and study design

All patients diagnosed with HCC were collected retrospectively from The Third Affiliated Hospital of Kunming Medical University between January 2020 and September 2022. All methods and experiments were performed in accordance with relevant guidelines and regulations. Approval from the ethics committee of the third affiliated hospital of Kunming Medical University was obtained. Every patient has signed an informed consent.

**Figure 1:**
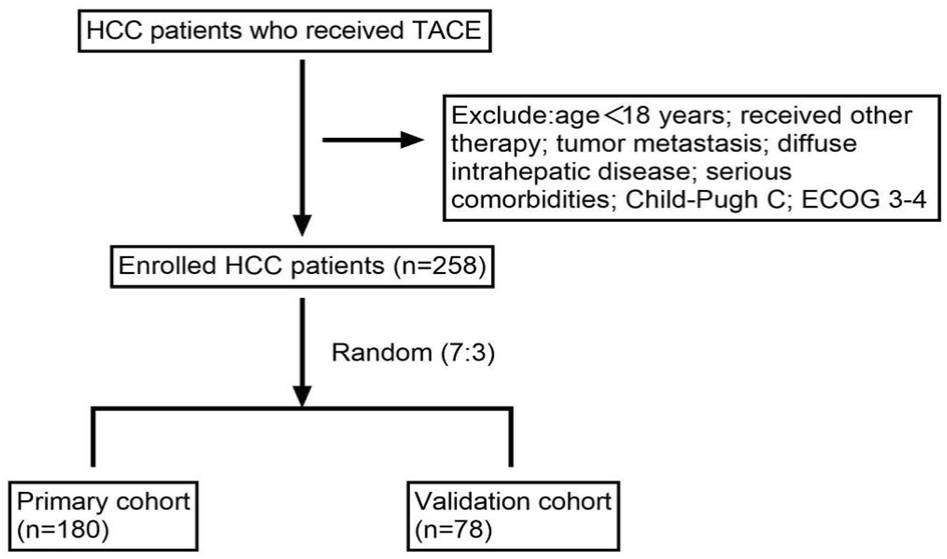
Flow diagram of the study selection process.

**Figure 2:**
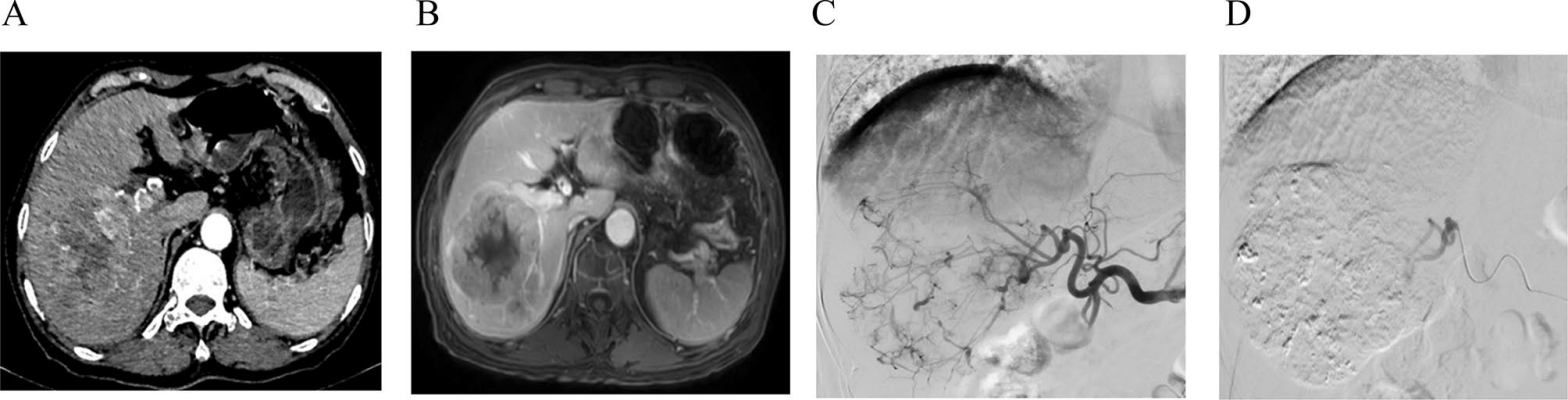
Example of the tumor condition and the procedure of TACE. (**A**, **B**) CT and MRI showed a heterogeneously enhanced tumor with a size of 10.5 cm × 8.4 cm located in the right posterior lobe of the liver; (**C**) angiography presented that the staining of tumor vasculature was found in the right liver lobe; (**D**) Iodized oil was deposited in the tumor and the feeding artery of tumor was obliterated after embolization.

Inclusion criteria to screen for suitable patients were set as follows: (1) at least 18 years of age with HCC diagnosis by computerized tomography (CT), magnetic resonance imaging (MRI), or alpha-fetoprotein (AFP) examination, (2) patients who lost the opportunity of radical surgery (3) patients who have not received other treatments such as ablation, chemotherapy, and radiotherapy, (4) patients who had no nausea, vomiting or pain within 24h before TACE and had not used antiemetic or painkillers, and (5) patients with Child–Pugh classification A or B. The exclusion criteria were: (1) age < 18 years, (2) received other therapy within 3 days after TACE, such as liver resection, ablation, chemotherapy, targeted or immune therapy; (3) patients with tumor metastasis, (4) patients with diffuse intrahepatic lesions; (5) patients with serious comorbidities, such as blood circulation disorders and respiratory insufficiency, especially when combined with respiratory or cardiac failure, and (6) patients with Child–Pugh classification C or ECOG 3–4. Finally, eligible patients were included in the study, and they were randomized to the primary cohort and validation cohort using the R package “caret ”. Then, patients in the primary cohort were categorized into the PES cohort and the non-PES cohort based on the presence or absence of PES (Fig. 1). The operations of all patients were performed by senior interventional physicians, and super-selective embolization was performed (Fig. 2).

### Study variables

**Figure 3:**
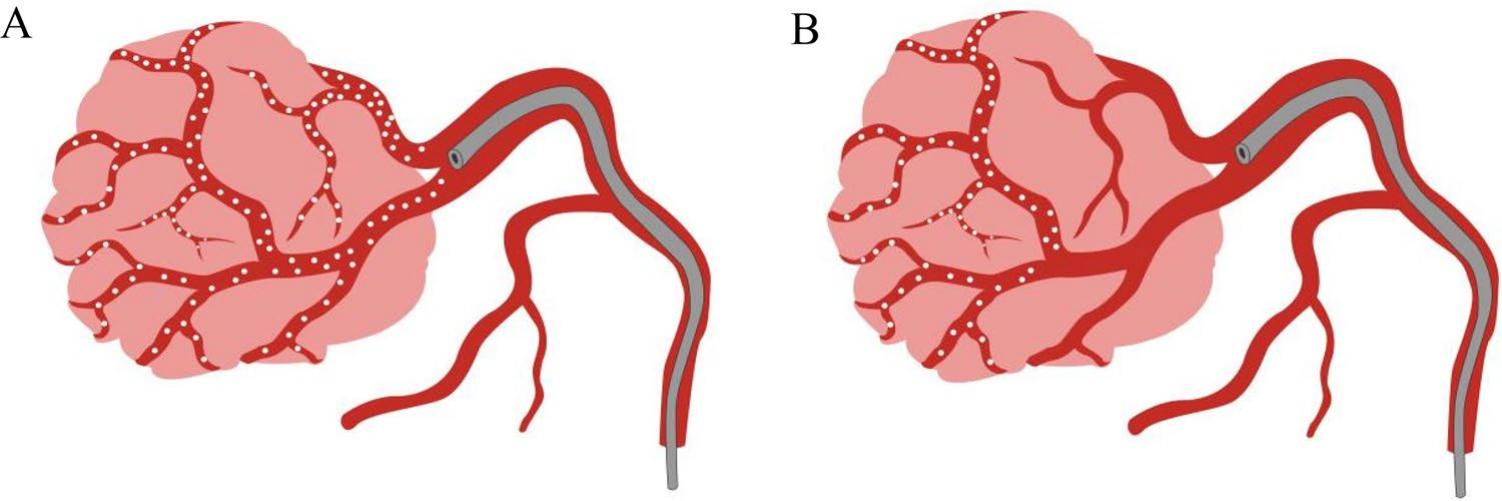
Schematic illustration for complete embolization (**A**) and incomplete embolization (**B**).

From the cohorts, data of the following variables were collected, including demographics (age and sex), body mass index (BMI), Eastern Cooperative Oncology Group (ECOG), liver cirrhosis, Child–Pugh class, Barcelona Clinic Liver Cancer (BCLC) staging, types of TACE, embolization degree, tumor number, diameter of the largest tumor, chemotherapeutic drugs, and operation time. The chief endpoint was PES, defined as a post-inflammatory clinical syndrome within three days after the intervention, such as fever and upper quadrant abdominal pain with or without nausea and vomiting. In our study, ECOG was classified into two subgroups: 0–1 and 2 points. TACE types were grouped into two subgroups: Conventional TACE (C-TACE) and drug-eluting bead TACE (D-TACE). In addition, the embolization degree was divided into incomplete embolization or complete embolization. The endpoint of embolization was defined as the flow rate of embolic fluid in the tumor-feeding artery not emptied within 3–4 cardiac cycles, or the supplying artery is in the shape of a “trunk branch.” When the embolization endpoint was achieved, or the embolization degree of the target vessel was greater than 95%, it was regarded as complete embolization, otherwise it is called incomplete embolization (Fig. 3). Then, based on preoperative imaging, the tumor number was organized into two subgroups based on preoperative imaging evaluation: 1 lesion, 2–4 lesions, and ≥ 5 lesions. Chemotherapeutic drugs were also assigned into two subgroups: single drug and multiple drugs.

### Statistical analyses

In this study, univariate analysis was performed to identify risk factors for a specific outcome. Categorical variables were analyzed by the Chi-squared or Fisher’s exact test, while continuous variables were evaluated by the student’s t-test, with P < 0.05 being statistically significant.

The identified risk factors were then used to construct a multivariate logistic regression model that estimated odds ratios for the outcome of interest. Then, a nomogram was constructed using the results of the logistic regression analysis, to predict PES incidence rates at 1, 2, and 3 days. The performance of the nomogram was evaluated using the concordance index (C-index), calibration curves, and receiver operating characteristic (ROC) curves. Larger the C-index represented more accuracy of prognostic prediction^9^. Concordance between actual and predicted PES occurrence probability was measured using calibration curves and 1000 bootstrap resamples.

The total points for eligible patients from the third affiliated Hospital of Kunming Medical University were calculated based on the scores assigned to each variable on the nomogram. Patients were classified into low- and high-risk groups based on their total score on the nomogram. Finally, the clinical usefulness of the nomogram in predicting PES occurrence was evaluated by plotting decision curves using the ggDCA package. All statistical analyses were performed using the R software (version 4.0.5) and SPSS software (version 21.0). A P-value < 0.05 was considered statistically significant.

### Ethics approval and consent to participate

The research was approved by the ethics committee of the third affiliated hospital of Kunming Medical University

## Results

### Patient characteristics

The enrolled patients were randomly assigned to the primary cohort (n = 180) and validation cohort (n = 78) based on the ratio of 7:3. As illustrated in Table 1, according to the occurrence of PES after TACE, patients in the primary cohort were categorized into the PES group (n = 106) and the non-PES group (n = 74). Among these patients, 96 (90.5%) males and 10 (9.5%) females were in the PES group and 63 (85.1%) males and 11 (14.9%) females were in the non-PES group. The average age of patients was 57.29 ± 9.84 years in the PES group and 59.20 ± 10.20 years in the non-PES group. Most patients in both groups were reported as Child–Pugh Class A and BCLC Staging B. The majority of patients did not have surgery and lymph node dissection. In the PES group, D-TACE were performed in 55 (51.8%) patients, and multiple chemotherapeutic drugs were used in the majority of patients. After analysis, these differences between the two groups were presented for ECOG, types of embolic agents, embolization degree, and tumor number (*P* < 0.05). In the primary cohort, the main symptom of most patients with PES was fever with abdominal pain (30.2%), followed by abdominal pain with vomiting (19.8%) and fever (18.9%). The symptoms of a few patients with PES were mainly nausea or vomiting (7.5%) (Table 2).

**Table 1:** Patient demographics and clinicopathologic characteristics in the primary cohort.

Demographic or characteristic	PES cohort n = 106 (100%)	Non-PES cohort n = 74 (100%)	X^2^/t	P-value
Age, years	57.29 ± 9.84	59.20 ± 10.20	− 1.262	0.209
Sex, N(%)			1.247	0.264
Male	96 (90.5%)	63 (85.1%)		
Female	10 (9.5%)	11 (14.9%)		
BMI, kg/m^2^	22.36 ± 3.04	22.45 ± 3.074	− 0.192	0.848
ECOG			9.249	< 0.001
0–1	59 (55.7%)	62 (83.8%)		
2	47 (44.3%)	12 (16.2%)		
Liver cirrhosis			0.054	0.861
No	52 (49.1%)	35 (47.3%)		
Yes	54 (50.9%)	39 (52.7%)		
Child–Pugh class			0.088	0.767
A	90 (84.9%)	64 (86.5%)		
B	16 (15.1%)	10 (13.5%)		
BCLC staging			3.286	0.070
B	78 (73.6%)	45 (60.8%)		
C	28 (26.4%)	29 (39.2%)		
TACE types			4.167	0.041
C-TACE	51 (47.2%)	47 (63.5%)		
D-TACE	55 (51.8%)	27 (36.5%)		
Embolization degree			20.481	< 0.001
Incomplete	30 (28.3%)	46 (62.2%)		
Complete	76 (71.7%)	28 (37.8%)		
Tumor number, n			6.353	0.042
1	40 (37.7%)	41 (55.4%)		
2–4	57 (53.8%)	26 (35.1%)		
≥ 5	9 (8.5%)	7 (9.5%)		
Tumor diameter, cm	7.30 ± 3.94	8.50 ± 4.18	− 1.952	0.052
Chemotherapeutic drugs			0.319	0.572
Single drug	14 (13.2%)	12 (16.2%)		
Multiple drug	92 (86.8%)	62 (83.8%)		
Operation time, min	87.13 ± 30.42	80.72 ± 24.95	1.493	0.137

BMI, body mass index; ECOG, Eastern Cooperative Oncology Group.

**Table 2:** Main symptoms of patients with post-embolization syndrome in the primary cohort.

Main symptoms	Frequency (%)
Fever	20 (18.9%)
Abdominal pain	12 (11.3%)
Nausea/vomiting	8 (7.5%)
Fever + abdominal pain	32 (30.2%)
Nausea/vomiting + abdominal pain	21 (19.8%)
Fever + abdominal pain + nausea/vomiting	13 (12.3%)

### Independent predictors of post-embolization syndrome

**Figure 4:**
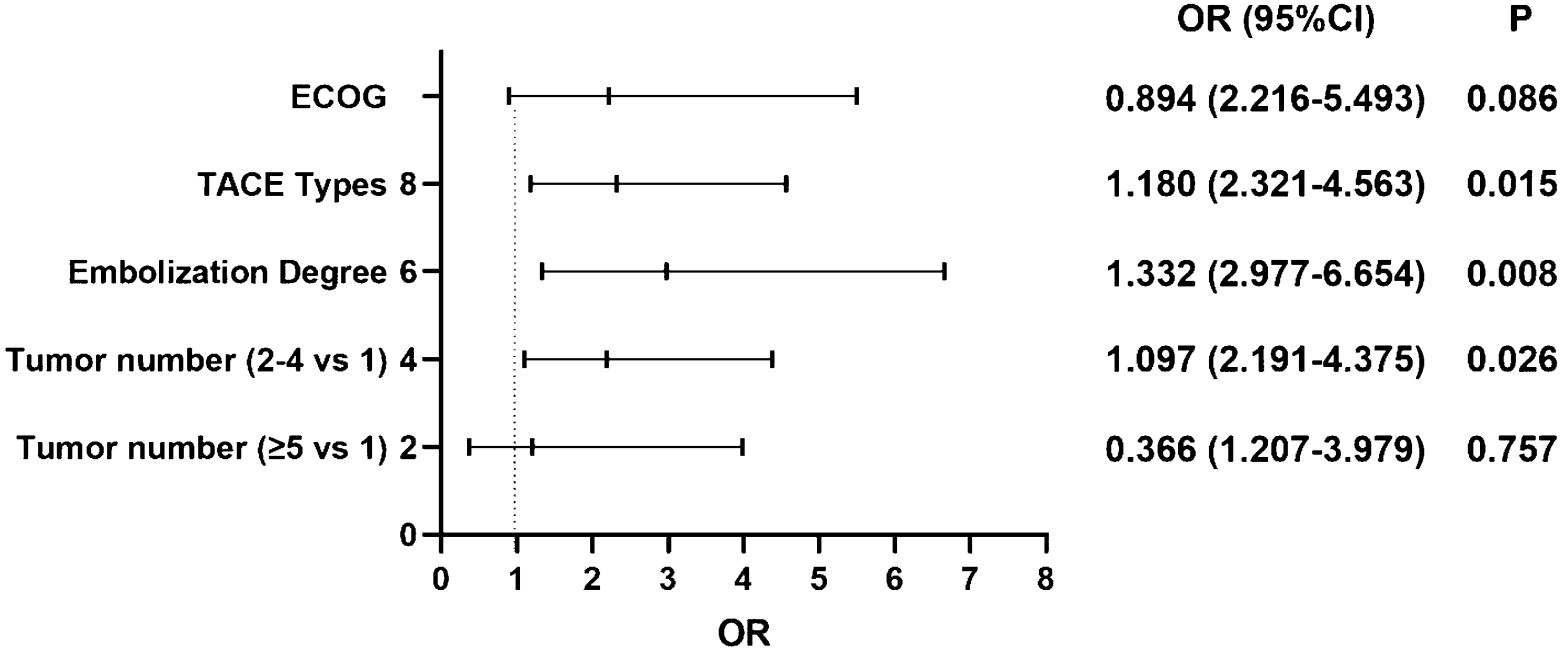
Logistic regression model analysis of post-embolization syndrome in the primary cohort.

After univariate analysis in the primary cohort, four variables were enrolled in the multivariable logistic regression analysis. The independent predictors of PES were D-TACE [OR 1.180 (2.321–4.563); *P* 0.015], complete embolization [OR 1.332 (2.977–6.654); *P* 0.008], and multiple lesions [OR 1.097 (2.191–4.375); *P* 0.026] (Fig. 4).

### Prognostic nomogram for post-embolization syndrome

**Figure 5:**
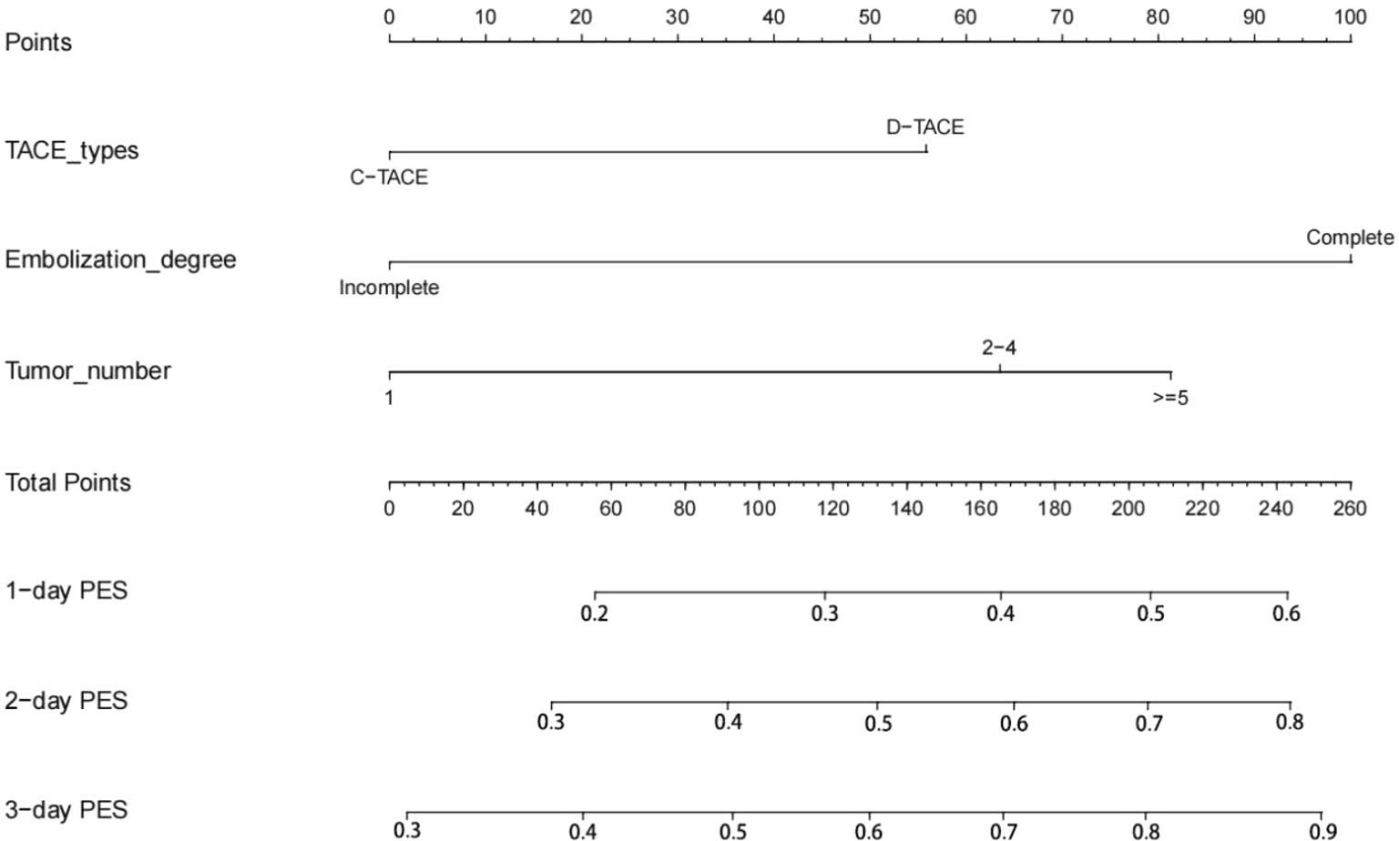
Predict nomograms for the incidence of post-embolization syndrome in the primary cohort.

According to the results of logistic regression analyses in the primary cohort, a nomogram integrating all the vital independent factors was built to predict 1-, 2-, and 3-day PES. This model indicated that embolization degree was the largest impact on prognosis, followed by types of tumor number, and embolic agents. The specific scoring system of the nomogram is shown in Fig. 5.

### Nomogram validation and risk stratification

**Figure 6:**
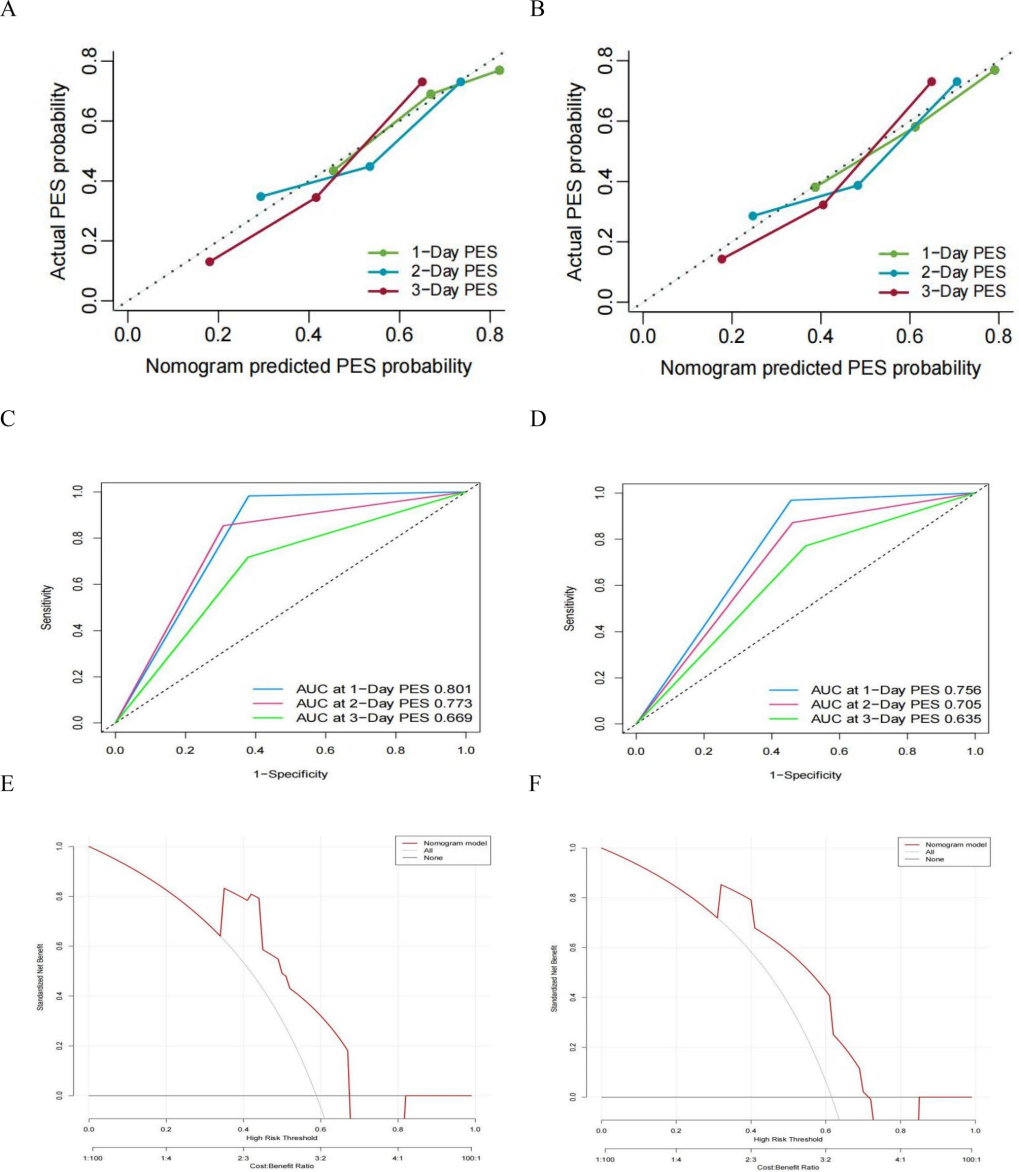
The calibration curves of the nomograms for post-embolization syndrome (PES) prediction at 1-, 2- and 3-day in primary cohort (**A**) and validation cohort (**B**). The ROC curve for PES at 1-, 2- and 3-day in primary cohort in primary cohort (**C**) and validation cohort (**D**). The clinical decision curve in primary cohort (**E**) and validation cohort (**F**).

The internal validation illustrated that the nomogram enables accurate predictions of PES with a C-index of 0.713 (95%CI = 0.685–0.732). Analogously, the C-index referred to 0.703 (95%CI = 0.680–0.725) in the external validation. As indicated in the calibration plots, an optimal consistency existed between the predicted PES and the actual PES for the 1-, 2-, and 3-day PES in the cohort. Moreover, ROC curves in primary cohort shows that 1-Day PES (AUC = 0.801) possessed the highest predictive accuracy compared with 1-Day PES (AUC = 0.773) and 3-Day PES (AUC = 0.669). Finally, Decision curve analysis further demonstrated the high net benefit and clinical practicability of this nomogram (Fig. 6). Besides, The total score of each patient was calculated using the R software (4.0.5, http://www.Rproject.org) based on the established logistic analysis, and the best cutoff value was selected. We classified the PES of the primary cohort according to the above method into: the risk score of the low-risk group for PES in the range of 59–114, and the risk score of the higher risk group in the range of 125–190.

## Discussion

TACE, as a commonly used treatment for HCC patients, has been proven to provide a survival benefit for those with advanced or unresectable diseases^10^. Although TACE is a relatively safe and effective procedure, it is always accompanied by PES, a self-limiting inflammatory syndrome related to the embolization itself or chemical drugs given. A recent report has shown that about 75% of the patients who underwent TACE developed PES, which was a relatively high rate of complications^11^. PES is mainly caused due to the embolization of a certain volume of liver tumor and normal liver tissue during TACE procedure, resulting in local liver tissue necrosis and edema, leading to a series of symptoms such as liver pain and heat absorption of necrotic tissue. In our study most patients with PES mostly had symptoms of abdominal pain with fever (30.2%). The side effects of chemotherapy drugs used in TACE treatment can lead to loss of appetite, nausea and vomiting, inadequate intake, and loss of sodium and water. The destruction of a large number of hepatocytes and the decrease in bile secretion, affects the digestion and absorption of food and further aggravates the symptoms of anorexia and nausea. Some patients with PES in the primary cohort also exhibited symptoms of nausea and vomiting. The occurrence of PES seriously influences the quality of life in patients and may further cause poor prognosis to a certain extent. Mason et al. reported twice as high mortality risk of patients with PES after TACE as that of patients without PES^12^. Similarly, several studies also presented higher mortality in patients who underwent TACE and developed PES^13,14^. Therefore, it is extremely important to effectively predict and control PES. The incidence of PES after TACE has been reported differently in the related reports^15,16^, which may be related to different general conditions, intraoperative use of embolic agents, and methods of data collection from TACE patients included in different literature. In this study, 180 patients were included, of which 106 cases (58.89%) developed PES. In our predictive model, D-TACE, complete embolization, and multiple lesions are significant predictors of PES, while age, sex, BMI, liver cirrhosis, Child–Pugh class, BCLC Staging, ECOG, diameter of the largest tumor, chemotherapeutic drugs and operation time were insensitive to PES and were not statistically significance.

In one recent study, patients with an ECOG score of 2 are more likely to develop PES than patients with a score of 0–1^17^. The reason may be that patients with an ECOG score of 0–1 have better mobility than patients with a score of 2, which led to a lower probability of postoperative PES. However, our results are in contrast with the conclusion reached by Gao et al. ECOG was not selected as a protective factor for PES in patients who received TACE in our study, which suggests that no clear relationship exists between PES and physical fitness. Besides, tumor number was a significant predictor of PES (*P* < 0.05). Compared with a single focus, multiple lesions mean a greater tumor load. This will lead to a widespread distribution of intraoperative chemotherapeutic drugs and embolic agents, causing a more severe inflammatory response, and thus, making the patient more prone to induce pain. Khalaf et al.^15^ also proposed that a large tumor burden was identified as a strong predictor for HCC patients who developed PES after TACE. Moreover, a recent study reported that HCC patients with bilobar tumor distribution are more likely to develop PES than patients with single tumor distribution^17^, which further indicates that tumor load has an important role in the occurrence of PES.

The process of embolization also became a non-negligible factor affecting postoperative PES. We also found that HCC patients with a D-TACE embolization are more likely to develop PES than patients who received C-TACE (*P* < 0.05). Previous studies^18,19^ have found that compared with traditional lipiodol, drug-loaded microspheres were more effective in the treatment of HCC, but whether different types of microspheres as embolic agents during operation would bring different adverse reactions has not been discussed. This can be explained by the fact that D-TACE patients with a large chemotherapeutic drug load may have a higher probability of inducing PES. A study has shown that the continuous release of chemotherapeutic agents is able to elicit a stronger inflammatory response, causing a more pronounced proinflammatory stimulus^20^. Thus, HCC patients who use drug-loaded microspheres as embolic agents during interventional operation may need more nursing of analgesia and antiemetic. In addition, compared with incomplete embolization, complete embolization was accepted as a risk factor for PES (*P* < 0.05). It can lead to the blockage of tumor-feeding arteries, resulting in liver tissue damage and episodes of severe pain. Yang et al.^21^ also draw a similar conclusion in their research.

A nomogram that can provide access to predict events accurately by a simple modeling graph has been developed and extensively accepted^22,23^. With the advent of the era of precision medicine, individualized treatment has become more meaningful and promising. In the past, some invasive predictive models for the prognosis of HCC have been developed^24,25^, but these models are often complex, costly, and their clinical practicability is relatively poor. Thus, non-invasive predictive models based on purely clinical indicators have more clinical value and are more easily applicable in clinical practice. Jiao Hu et al.^26^ conducted a multicenter retrospective real-world study based on patient imaging and peripheral blood data to explore the efficacy of three neoadjuvant chemotherapy in patients with myometrial invasive bladder cancer and the possible factors affecting the efficacy. They could develop and validate an efficient non-invasive prediction model that included preoperative clinical features to identify candidates for neoadjuvant combination therapy.

Recently, several nomograms have been established for prognosis of patients with HCC treated by TACE^27,28^. However, concerning the nomogram for PES after the treatment of TACE, few studies are available. Based on the results of logistic regression analyses, we first tried to construct a nomogram for exploring the probability of 1-, 2-, and 3-day PES in HCC patients who received TACE. The C-index and calibration curve were also performed for the predictive accuracy of this model on our cohort, and the values were 0.713 and 0.703, respectively, which presents optimal consistency between predicted and actual PES. Consistent with previous research^13^, the nomogram identified that embolization degree was the largest impact on the occurrence of PES, and types of embolic agents also presented a moderate influence on PES. The total risk score for each patient can be obtained by summing the scores of all variables, and the corresponding incidence of PES can be inferred. For example, if an HCC patient with 2–4 tumors received D-TACE of complete embolization, the scores for each factor were 2–4 tumors (62 points), D-TACE (55 points), and complete embolization (100 points). The total score was, therefore, approximately 217 points. Our model predicted a 50% probability of developing PES for 1 day, a 70% probability of developing PES for 2 days, and an 80% probability of developing PES for 3 days. This model can, therefore, prospectively predict patients who could develop PES after TACE, which will help clinicians to provide preventive interventions in advance for those patients with high risk of PES. Moreover, such a model also helps doctors to identify which patients can withstand early discharge and which patients need to stay in hospital because of the risk of postoperative PES. This effectively adjusts the average length of hospital stay and has certain socioeconomic value.

Several potential limitations should be acknowledged in our study. First, because of the retrospective nature, selection bias could occur. Second, some pain of non-PES sources could not be clearly distinguished, which may cause to over-quantify PES-related pain in our study. Finally, a limited number of cases for establishing a predictive model became a statistical challenge. Further prospective, multicenter, large-sample studies are still required in the future.

Taken together, types of embolic agents, embolization degree, and tumor number were considered as predictors of PES after the TACE. Among them, embolization degree as a significant predictor for PES showed a stronger influence on predicting PES. Besides, we also constructed and validated a predictive nomogram based on multivariable logistic regression analysis predicting PES for HCC patients who underwent TACE, which could provide an individualized PES prediction and guidance to appropriate therapeutic strategy for patients with HCC.

## Data Availability

The datasets used during the current study are available from the corresponding author upon reasonable request.
